# Factors associated with emergency department visit within 30 days after discharge

**DOI:** 10.1186/s12913-016-1439-x

**Published:** 2016-05-25

**Authors:** Chuan-Lan Wang, Shih-Tan Ding, Ming-Ju Hsieh, Chin-Chung Shu, Nin-Chieh Hsu, Yu-Feng Lin, Jin-Shing Chen

**Affiliations:** Department of Nursing, National Taiwan University Hospital, Taipei, Taiwan; Department of Traumatology, Hospital Medicine Group, National Taiwan University Hospital, #7, Chung-Shan South Road, Taipei, 100 Taiwan; Department of Emergency Medicine, National Taiwan University Hospital, Taipei, Taiwan; Graduate Institute of Clinical Medicine, College of Medicine, National Taiwan University, Taipei, Taiwan; College of Medicine, National Taiwan University, Taipei, Taiwan; Department of Surgery, National Taiwan University Hospital, Taipei, Taiwan

**Keywords:** Emergency department visit, Hospital medicine, Post-discharge transitional care, Readmission, Taiwan

## Abstract

**Background:**

Post-discharge care remains a challenge because continuity of care is often interrupted and adverse events frequently occur. Previous studies have focused on early readmission but few have investigated emergency department (ED) visit after discharge.

**Methods:**

This retrospective observational study was conducted between April 2011 and March 2012 in a referral center in Taiwan. Patients discharged from the general medical wards during the study period were analyzed and their characteristics, hospital course, and associated factors were collected. An ED visit within 30 days of discharge was the primary outcome while readmission or death at home were secondary outcomes.

**Results:**

There were 799 discharged patients analyzed, including 96 (12 %) with an ED visit of 12.4 days post-discharge and 111 (14 %) with readmissions at 13.3 days post-discharge. Sixty patients were admitted after their ED visit. Underlying chronic illnesses were associated with 72 % of ED visits. By multivariate analysis, Charlson score and the use of naso-gastric tube were independent risk factors for ED visit within 30 days after discharge.

**Conclusions:**

Early ED visit after discharge is as high as 12 %. Patients with chronic illness and those requiring a naso-gastric tube or external biliary drain are at high risk for post-discharge ED visit.

## Background

Post-discharge care remains a challenge, especially for the elderly and those with underlying co-morbidities [1–3]. About 10 % of discharged patients have new or worsening symptoms within days to weeks [4] and the readmission rate is high, with a 30-day re-hospitalization rate of 15–20 % in literature [2, 5–7]. Factors associated with readmission include age, sex, race, and length of hospital stay, and number of previous hospitalizations [2]. Several possible reasons for readmission include instability of chronic disease and insufficient communication among physicians [8].

Aside from readmission, emergency department (ED) utilization is another adverse event after discharge, representing patient’s instability after discharge [7, 9–11]. There is a high proportion of hospitalization after ED visit, leading to higher costs [12–14]. Thus, post-discharge transitional care such as phone-contact, home visits, and integrated strategy are needed to identify those at high-risk for ED visit after discharge [15–18].

Although risk factors for early readmission are well evaluated, predictors of post-discharge ED visit by general medical patients have rarely been investigated. This retrospective study aimed to identify the causes, time course, and risk factors associated with ED visit after discharge from a general ward in a Taiwan hospital.

## Methods

### Study subjects

This retrospective observational study was conducted at the National Taiwan University Hospital, a tertiary-care referral center in Taiwan, from April 1, 2011 to March 31, 2012. All patients aged >20 years admitted to the general wards were screened. Index hospitalization was defined as the first admission during the study period. Those who were discharged alive were identified. Those who died during the index hospitalization, went home for anticipated dying, or were transferred to other departments or hospitals were excluded. The Institutional Review Board of the hospital’s Research Ethics Committee approved the study protocol. Written informed consent was waived.

### Data collection

The patient characteristics, laboratory data, courses of index hospitalization, diagnoses, and dates of ED visits and readmissions were recorded using the hospital’s electronic medical records. Information on the date of death at home or living status was also acquired from records or from a routine survey of patients within 30 days after discharge. A unified recording form with a default option for selection to prevent ambiguous data coding was used.

The Charlson co-morbidity index and Barthel index were calculated as in previous studies [19, 20]. Underlying malignancy was defined as active cancer without mention of cure or remission. The primary care physician was defined as the doctor who was visited by the patient three or more times within one year prior to the index hospitalization [21]. A five-level triage system (1, resuscitation; 2, emergency; 3, urgent; 4, less urgent; and 5, not urgent) was used in the ED [22]. Artificial tube/catheter included naso-gastric tube, tracheostomy tube, draining tube, Foley catheter, and catheter for dialysis. Information was obtained from the medical records.

The clinical course and clinical diagnosis of each ED visit and readmission were reviewed. The cause of the ED visit was determined from the medical records. The causes of post-discharge adverse events were categorized by a nurse and a hospitalist, who independently decided if the causes were the same as those of the index hospitalization or if the causes were associated with chronic illness or malignancy. Any discrepancy was settled by consensus.

### Outcome measurement and statistical analysis

The primary endpoint was the first ED visit within 30 days after discharge. The secondary endpoints included readmission and mortality within 30 days after discharge. Inter-group differences were compared using independent *t* test for numerical variables and chi-square test for categorical variables. After using the forward conditional selection method of all clinically relevant factors, multivariate Cox proportional hazard regression was used to identify factors associated with ED visit or adverse events within 30 days after discharge.

For multivariate models, patients were censored for endpoints of ED visit, readmission, or mortality. No one was lost to follow-up within 30 days. In terms of missing data (4 [0.5 %] in hemoglobin and 71 [8.9 %%] in discharge Barthel score), these were categorized as “unknown data” to avoid case loss in the multivariate model. Statistical significance was set at a two-sided *p* < 0.05. All analyses were performed using the SPSS (Version 15.0, Chicago, IL).

## Results

Of the 1028 patients in the general medical wards who were screened, 1012 (98.4 %) were admitted from the ED. During the index hospitalization, 124 were transferred to other departments for further treatment, 13 were transferred to other hospitals, and 92 died before discharge or went home for the dying process. A total of 799 patients were included in the final analyses, including 753 (94 %) who returned home or those who were brought to a nursing home after discharge.

**Fig. 1:**
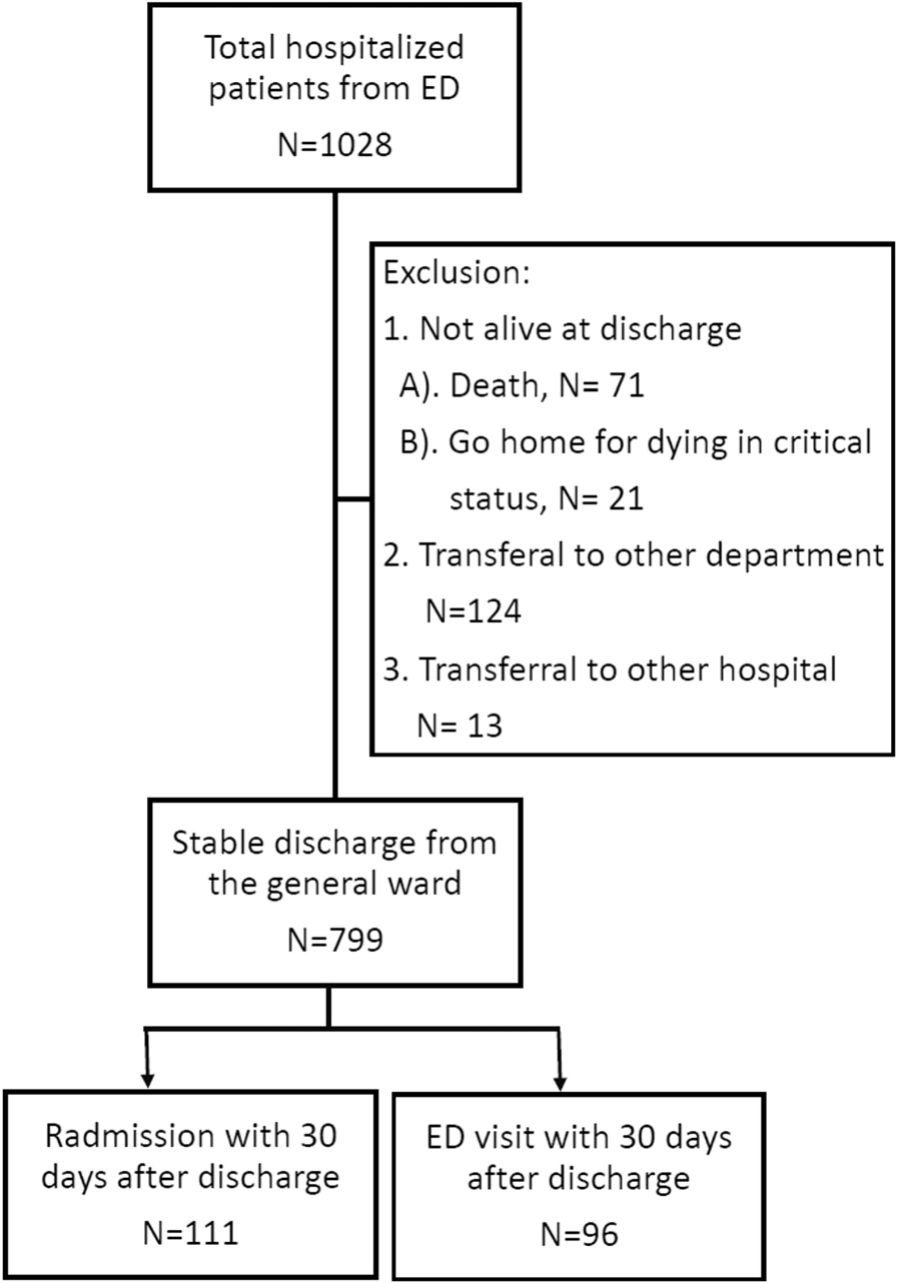
Flow chart of patient enrollment

During the 30-day period after discharge, 96 (12 %) patients visited the ED, 111 (14 %) were readmitted, and two died at home (Fig. 1). The mean age (71.7 vs. 69.9 years) and sex (male: 45 % vs. 49 %) were similar between patients who visited the ED and those who did not (Table 1). The Charlson co-morbidity index scores were higher in patients with ED visit (age-unadjusted, 3.1 ± 2.5 vs. 2.4 ± 2.5; *p* = 0.013) and in those with readmission (age-unadjusted: 3.0 ± 2.5 vs. 2.4 ± 2.5, *p* = 0.023) within 30 days post-discharge. The age-adjusted Charlson score was only significantly higher in patients who visited the ED (5.8 ± 2.8 vs. 4.9 ± 3.0, *p* = 0.006).

**Table 1:** Clinical characteristics of patients according to readmission or emergency department (ED) visit within 30 days post-discharge

	Patients with ED visit (*n* = 96)	Patients without ED visit (*n* = 703)	Patients readmitted (*n* = 111)	Patients, not readmitted (*n* = 688)
Age, years	71.7 ± 14.8	69.9 ± 16.7	69.1 ± 15.2	70.3 ± 16.7
Sex, male	43 (45)	347 (49)	51 (46)	339 (49)
Charlson Co-morbidity index Unadjusted	3.1 ± 2.5*	2.4 ± 2.5	3.0 ± 2.5*	2.4 ± 2.5
Age-adjusted	5.8 ± 2.8**	4.9 ± 3.0	5.4 ± 2.8	5.0 ± 3.0
Primary care physician, presence	73 (76)	477 (67)	87 (78)*	463 (67)
Length of hospital stay, days	11.9 ± 6.4	10.5 ± 9.2	12.7 ± 10.4*	10.3 ± 8.7
Artificial tube/catheter				
Naso-gastric tube	25 (26)**	101 (14)	22 (20)	104 (15)
Foley Catheter	7 (7)	41 (6)	8 (7)	40 (6)
Tracheostomy	4 (4)	18 (3)	5 (5)	17 (3)
Percutaneous biliary drain	3 (3)*	6 (1)	3 (3)	6 (1)
Initial^a^ hemoglobin, g/dL	10.8 ± 2.3*	11.3 ± 3.2	10.7 ± 2.2*	11.4 ± 3.3
Barthel index score				
At admission	50.1 ± 34.6	56.6 ± 36.3	53.6 ± 35.9	56.2 ± 36.2
At discharge^a^	54.0 ± 41.9	62.9 ± 40.5	58.8 ± 40.8	62.3 ± 40.7
Wound requiring dressing	13 (14)	72 (10)	13 (12)	72 (11)

Data are no. (%) or mean ± standard deviation unless otherwise indicated

Statistical significance was compared between readmitted and non-readmitted patients or patients with and those without ED visit

*Means 0.01 < *p* < 0.05; ** means *p* < 0.01

^a^Missing hemoglobin in four patients without post-discharge adverse event and missing discharge Barthel score in 71 (one in the group with adverse event)

The Barthel index for daily activity, proportion of primary care physician, and presence of wound requiring dressing were similar between patients who visited the ED and those who did not. Patients who visited the ED also had higher percentages of requiring naso-gastric tube or biliary tract drainage (26 % vs. 14 %; *p* = 0.003 and 3 % vs. 1 %; *p* = 0.048, respectively).

Regarding early (within 30 days) post-discharge adverse events, patients visited the ED around 12.4 days after discharge and were readmitted around 13.3 days after discharge (Table 2). There were 62 (64.5 %) and 65 (58.5 %) patients who visited their primary care physician before their ED visit and readmission, respectively. Thirty-three (34 %) visited the ED within one week after discharge. Among the patients with ED visits, the average triage level (±standard deviation [SD]) was 2.46 ± 0.64.

**Table 2:** Nature of readmission and emergency department visit within 30 days post-discharge

	Patients readmitted (*n* = 111)	Patients with ED visit (*n* = 96)
Days after discharge	13.3 ± 8.0	12.4 ± 8.3
Cause category		
Same illness as last admission	56 (50)	66 (69)
New illness	55 (50)	30 (31)
Chronic illness association	69 (62)	69 (72)
Malignancy related	31 (28)	28 (29)

Data are no. (%) or mean ± standard deviation unless otherwise indicated

**Fig. 2:**
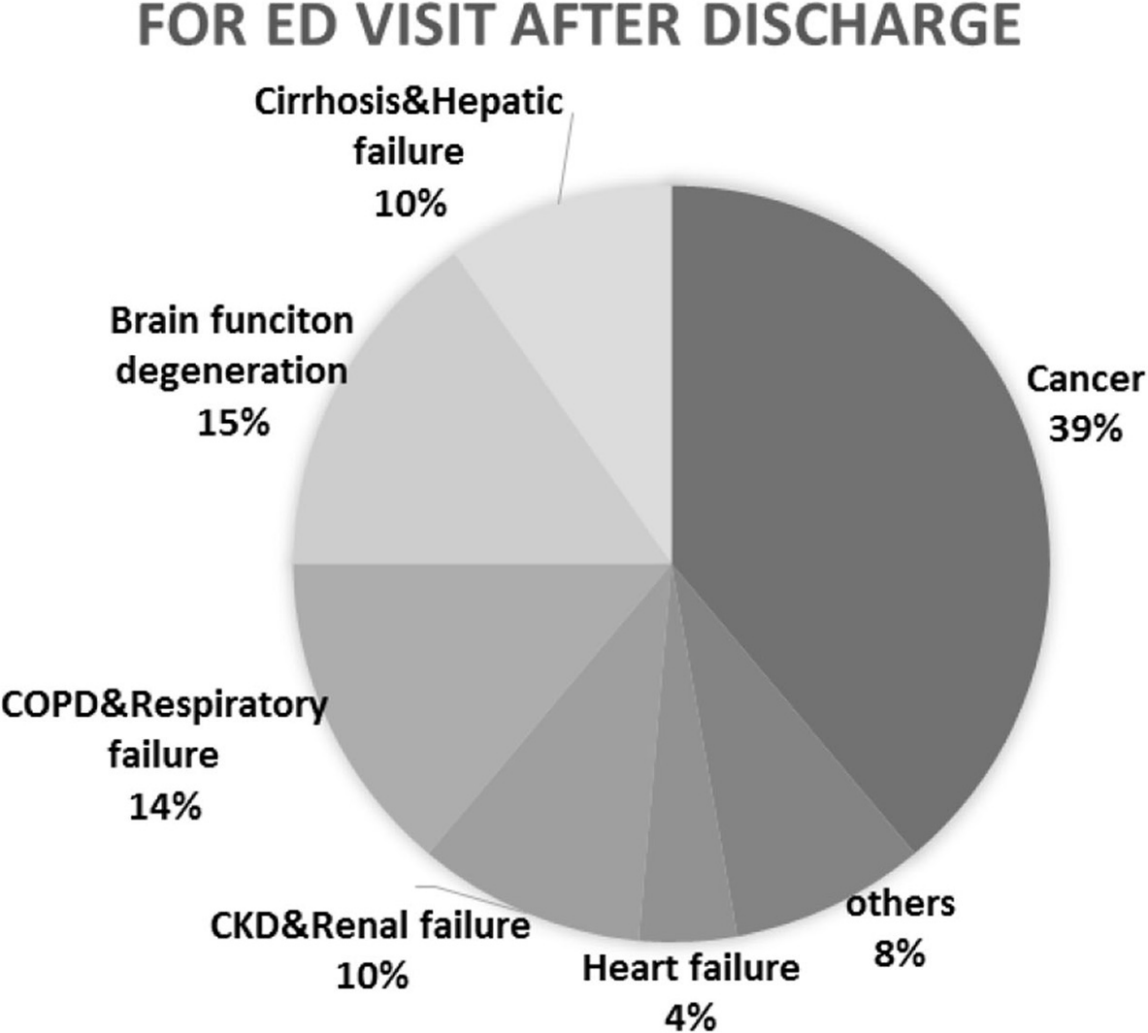
Category of chronic illnesses associated with emergency department (ED) visit post-discharge. CKD, chronic kidney disease; COPD, chronic obstructive pulmonary disease

The cause of the index hospitalization was also the main cause in 69 % of ED visits and in 50 % of readmissions. Underlying chronic illnesses were associated with 72 % of ED visits and 62 % of readmissions, while underlying malignancy and organ failure accounted for 39 and 53 % of ED visits, respectively, and 45 and 47 % of readmissions, respectively (Fig. 2).

Among patients with ED visits, 52 (54 %) were admitted from the ED while eight were discharged from the ED but returned and were admitted after the 2nd ED visit within the 30-day window. Those not admitted had a median ED stay of 8 h (inter-quartile range [IQR], 3.5–75.5 h).

In multivariate analysis (Table 3), the independent factors for ED visit within 30 days after discharge were non-adjusted Charlson co-morbidity score (Hazard Ratio [HR]: 1.108; 95 % confidence interval [CI]: 1.030–1.192), use of naso-gastric tube (HR: 2.081, 95 % CI: 1.315–3.293), and use of external biliary tract drain (HR, 4.191, 95 % CI, 1.298–13.261). Longer index hospitalization (HR, 1.014, 95 % CI, 1.02–1.026), age-adjusted Charlson co-morbidity index (HR, 1.067, 95 % CI, 1.012–1.124), and use of external biliary tract drain (HR, 4.158, 95 % CI, 1.679–10.296) were independent risk factors associated with adverse events, including ED re-visit, readmission, and death at home.

**Table 3:** Multivariate analysis of factors possibly associated with readmission or ED visit within 30 days post-discharge

Characteristics	For ED visit		For adverse events^a^	
	*p* value	HR (95 % C.I.)	*p* value	HR (95 % C.I.)
Age, years	0.959		0.391	
Sex, male	0.320		0.590	
Length of hospital stay, days	0.398		0.024	1.014 (1.002–1.026)
Charlson co-morbidity index				
Non-adjusted	0.006	1.108 (1.030–1.192)	0.532	
Age-adjusted	0.511		0.015	1.067 (1.012–1.124)
Barthel index score				
At admission	0.722		0.094	
At discharge	0.745		0.105	
Artificial tube/catheter	0.611		0.283	
Naso-gatric tube	0.002	2.081 (1.315–3.293)	0.067	
Urinary catheter	0.545		0.753	
Tracheostomy	0.897		0.502	
External drain for biliary tract	0.016	4.191 (1.298–13.261)	0.002	4.158 (1.679–10.296)
Wound needs dressing	0.569		0.616	

*Abbreviation: ED* emergency department, *CI* confidence interval

^a^Represents ED visit, readmission, or death at home within 30 days after discharge from index hospitalization

## Discussion

This retrospective study reviewed data of discharged patients and revealed that 12 % visited the ED in an average of 12.4 days after discharge, while 14 % were readmitted in 13.3 days. The same Illness during the index hospitalization was responsible for 69 % of ED visits and chronic illness correlated with 72 %. Malignancy and organ failure were the most common underlying co-morbidities. The Charlson co-morbidity index and use of naso-gastric tube or external biliary tract drain were independent predictors of ED visit within 30 days post-discharge. On the other hand, age-adjusted Charlson co-morbidity index, length of index hospitalization, and use of external biliary drain were associated with adverse events of ED visit, readmission, or death.

Early post-discharge ED visit was high (12 %) for patients with general medical illness and was similar to those of patients who underwent heart surgery (11.9 %) or colon resection for cancer (9.2 %) [23, 24]. Among patients on dialysis, ED visit was as high as 27 % within 30 days post-discharge [7].

The underlying co-morbidity may be one of the most important factors predicting post-discharge ED visit. A higher proportion of primary care physicians for readmitted patients also indicates that these patents have more chronic illness that require regular clinic visits before the index hospitalization. The high Charlson score is reasonable because it represents the complexity of the underlying disease and is important for readmission or other post-discharge adverse events [25–27].

In this study, age is not associated with readmission and ED visit. This is similar to findings of a previous report for readmission [26]. However, age-adjusted Charlson score is still a predictor of readmission and is indicative that age alone is less influential. A possible reason is that many discharged patients are middle-aged but have underlying cancer or liver cirrhosis in the study referral center. Furthermore, the Charlson score, without age adjustment, is associated with ED visit. Thus, more attention should be given to a high Charlson score even in young patients.

In a previous study for readmission-associated illness, organ failure and cancer are the most common disease conditions [28]. Similarly, in the present study, organ failure is considered the leading problem. If all of the different kinds of organ failure are taken together, organ failure ranks higher than cancer.

Artificial catheter or tube that requires long-term care has several clinical meanings for a discharged patient. First, it indicates clinical dysfunction, like when a naso-gastric tube is required for swallowing dysfunction. Second, it is associated with chronic illness, like an external biliary drain that is used for cancer-related obstructive jaundice. Third, higher care skills are needed for patients who require tubes/catheters. The skills of caregivers are important in post-discharge transitional care, especially for patients without self-care ability [13, 17, 29, 30]. As such, the use of a tube/catheter may be a basis for requiring high level of care skills, especially when the associated chronic illness is severe. For example, an external biliary drain is usually associated with advanced liver cancer and biliary tract obstruction. For discharged patients with a high Charlson score and who require external naso-gastric tube or external biliary drain, education of care skills and monitoring of disease stability may be key points for reducing post-discharge adverse events.

In this study, by multivariate analysis, the Barthel index of a patient’s daily living activity does not correlate with ED visit. This is possible because the Barthel index on admission and on discharge may change with time, according to disease course [31]. The problem associated with low Barthel index is the key point, like an artificial tube/catheter. Moreover, complications of underlying diseases may not be predicted by the Barthel index (e.g., variceal bleeding in a cirrhotic patients leading to acute changes that do not correlate with daily activity). The causes of such adverse events are multi-factorial.

The present study has several limitations. First, because it was performed in a tertiary referral center, patients might be more severely ill, with a higher proportion of cancer cases. This is important for generalizing to regional or district hospitals or to non-hepatitis prevalent areas. Furthermore, as a retrospective study, discharge education and planning are not unified. Information of medication compliance were not been obtained. Third, the number of patients with external biliary drain was small, so the 95 % confidence interval of hazards ratio for this risk was wide. We should interpret the data carefully. Lastly, post-discharge adverse events might be biased in methods of phone contact and chart review.

## Conclusions

Within 30 days after discharge, ED visit accounts for as high as 12 % in general medical patients. Patients with high Charlson co-morbidity score and those who use a naso-gastric tube or an external biliary drain are at high risk for post-discharge ED visit. Future studies for transitional care to minimize post-discharge adverse events are warranted.

## Abbreviations

CI, confidence interval; CKD, chronic kidney disease; COPD, chronic obstructive pulmonary diseaseED, emergency department; HR, hazard ratio
